# Tight DNA-protein complexes isolated from barley seedlings are rich in potential guanine quadruplex sequences

**DOI:** 10.7717/peerj.8569

**Published:** 2020-02-18

**Authors:** Tatjana Sjakste, Elina Leonova, Rudolfs Petrovs, Ilva Trapina, Marion S. Röder, Nikolajs Sjakste

**Affiliations:** 1 Genomics and Bioinformatics Group, Institute of Biology, University of Latvia, Riga, Latvia; 2 Faculty of Medicine, University of Latvia, Riga, Latvia; 3 Leibniz Institute for Plant Genetics and Crop Research, Gatersleben, Germany

**Keywords:** Deproteinisation-resistant DNA-protein complexes, Barley, GC-rich DNA, G-quadruplexes

## Abstract

**Background:**

The concept of chromatin domains attached to the nuclear matrix is being revisited, with nucleus described as a set of topologically associating domains. The significance of the tightly bound to DNA proteins (TBP), a protein group that remains attached to DNA after its deproteinization should be also revisited, as the existence of these interactions is in good agreement with the concept of the topologically associating domain. The work aimed to characterize the DNA component of TBP isolated from barley seedlings.

**Methods:**

The tight DNA-protein complexes from the first leaves, coleoptiles, and roots of barley seedlings were isolated by purification with chromatography on nitrocellulose or exhaustive digestion of DNA with DNase I. Cloning and transformation were performed using pMOSB*Blue* Blunt Ended Cloning Kit. Inserts were amplified by PCR, and sequencing was performed on the MegaBace 1000 Sequencing System. The BLAST search was performed using sequence databases at NCBI, CR-EST, and TREP and Ensembl Plants databases. Comparison to MAR/SAR sequences was performed using http://smartdb.bioinf.med.uni-goettingen.de/cgi-bin/SMARtDB/smar.cgi database. The prediction of G quadruplexes (GQ) was performed with the aid of R-studio library pqsfinder. CD spectra were recorded on a Chirascan CS/3D spectrometer.

**Results:**

Although the barley genome is AT-rich (43% of GC pairs), most DNA fragments associated with TBP were GC-rich (up to 70% in some fractions). Both fractionation procedures yielded a high proportion of CT-motif sequences presented predominantly by the 16-bp CC(TCTCCC)_2_ TC fragment present in clones derived from the TBP-bound DNA and absent in free DNA. BLAST analysis revealed alignment with different barley repeats. Some clones, however, aligned with both nuclear and chloroplast structural genes. Alignments with MAR/SAR motifs were very few. The analysis produced by the pqsfinder program revealed numerous potential quadruplex-forming sites in the TBP-bound sequences. A set of oligonucleotides containing sites of possible GQs were designed and ordered. Three of them represented the minus strand of the CT-repeat. Two were derived from sequences of two clones of nitrocellulose retained fraction from leaves and contained GC-rich motifs different from the CT motif. Circular dichroism spectroscopy revealed profound changes in spectra when oligonucleotides were incubated with 100 mM KCl. There was either an increase of positive band in the area of 260 nm or the formation of a positive band at 290 nm. In the former case, changes are typical for parallel G-quadruplexes and, in the latter, 3 + 1 structures.

**Discussion:**

The G-quadruplexes anchor proteins are probably involved in the maintenance of the topologically associated domain structure.

## Introduction

Recent achievements in studies of three-dimensional interactions between genes caused a significant re-evaluation of the nucleus structure in general. The traditional concept of chromatin domains attached to the nuclear matrix is being revisited. The nucleus is described as a set of topologically associating domains, and DNA-protein interactions develop within these domains (Razin & Vassetzky, 2017; Razin & Ulianov, 2017; Razin, Iarovaia & Vassetzky, 2014). The very existence of the nuclear matrix and specific matrix-attachment regions in DNA is denied (Hancock, 2004; Hancock, 2014; Razin, Iarovaia & Vassetzky, 2014). However, one cannot deny the existence of tight DNA-protein interactions. From this point of view, the significance of the so-called tightly bound to DNA proteins (TBP), a protein group that remains attached to DNA with covalent or non-covalent bonds after its deproteinization, (reviewed in Sjakste et al., 2009; Sjakste et al., 2012) should be also revisited as the existence of these interactions is in good agreement with the topologically associating domains concept.

Thus, the characterization of DNA sequences attached to the TBPs is of special interest. The TBP distribution in the genome is site-specific. They are enriched in several reiterated sequences (Pfütz, Gileadi & Werner, 1992; Werner & Neuer-Nitsche, 1989; Neuer-Nitsche, Lu & Werner, 1988). However, no common sequences have been found up until now. No significant similarities were also detected between several signal sequences of a given organism. It was concluded that either the site-specific binding of the non-histone polypeptides is induced by very different DNA sequences or signals were based on other characteristics than the basic DNA sequence (Pfütz, Gileadi & Werner, 1992). However, this conclusion seems to be precocious, as very few DNA fragments were sequenced and analyzed in the above experiments. Both the function of the TBPs and nature of DNA sequences involved in the tight complexes remain to be revealed.

We have performed a series of studies aimed at the evaluation of the functional role of the tight DNA-protein complexes in barley cells using the non-etiolated barley shoots as a model. The distribution of DNA complexes with TBP in the barley chromosome 1H was first studied by means of microsatellite analysis. The study revealed organ- and development-stage-specific changes in association with the different chromosomal sites with TBP (Sjakste et al., 2005). In dry seeds, the association of the markers with TBPs was random. The association became organ-specific after germination. Later, using the DNA microarray technique, we observed changes in the distribution of the TBP along with amylase genes during the seedling development: the sequences of amylase genes became associated with the TBPs after the onset of transcription of these genes (Sjakste et al., 2009). Developmental changes in polypeptide composition and enzymatic activities of the barley TBPs were also studied (Bielskiene et al., 2012). Evidently, the DNA sequences associated with TBPs played a significant role in the regulation of genome activity. These results encouraged us to extend the study of the specificity of DNA fragments associated with TBPs in different organs of barley seedlings. The goals of the present study were: (1) to clone the DNA attached to the TBPs using chromatography on nitrocellulose and exhaustive DNase digestion; (2) to sequence the TBP-attached DNA and perform bioinformatic analysis of the sequences; and (3) to analyze possibilities of the formation of unusual DNA structures in this DNA fraction and test the ability of the most important repeats to form quadruplexes experimentally.

## Materials and methods

### Chemicals

Tris base, ethidium bromide, agarose, Hind III/*λ* DNA digest, ethidium bromide (EBr), Na_2_EDTA, Na bisulfite, NaOH, LiCl, KCl, NaCl, and other inorganic salts were purchased from Sigma-Aldrich. Sodium dodecyl sulfate was supplied by Acros Organics. Buthoxyethanol, chloroform and isoamylic alcohol were obtained from Stanlab. Restrictases and Proteinas K were purchased from Fermentas.

### Plant material

The work was performed using the “Balga” barley cultivar plants. This cultivar was created by Latvian breeders. We used it to study the development-dependent changes in TBP complexes (Sjakste et al., 2009). For DNA extraction, we routinely used about 100 seedlings. To obtain the plant organs, grains were germinated; shoots were grown for 5 days at 30 °C temperature in the darkness. The first leaf, coleoptile and root were dissected and subsequently used for DNA extraction.

### Solutions

DNA extraction buffer: 100 mM TrisHCl, pH 7.5; 500 mM NaCl, 50 mM Na_2_EDTA, 1.25% SDS and 3.8 g/l Na bisulfite. Filtration buffer: 0.5 M KCl, 5 mM EDTA, 10 mM Tris- HCl, pH 7.4. Elution buffer: elution buffer 5 mM EDTA, 10 mM TrisHCl, pH 7.4. DNase buffer: 10 mM Tris–HCl, pH 7.6; 5 mM MgCl_2_. Folding buffer: 10 mM sodium phosphate and 0.3 mM EDTA, pH 7.1.

### DNA isolation

Bulk genomic DNA was extracted according to the previously described protocol of chloroform-isoamylic alcohol extraction (Plaschke, Ganal & Röder, 1995), with some modifications. Plant tissues were frozen in liquid nitrogen and ground with a mortar into a fine powder. The powder was suspended in 25 ml of the pre-warmed (65 °C) extraction buffer. The mixture was incubated for 30 min at 65 °C in the same buffer with occasional shaking. DNA was extracted with the same volume of chloroform/isoamilic alcohol mixture (24:1); the suspension was centrifuged, and the water phase was separated. RNA was precipitated with concentrated LiCl solution: the salt was added up to 4M and the mixture incubated for 1 h on ice. The sample was centrifuged to pellet the RNA. DNA was precipitated with 1 volume of butoxyethanol. The quality of DNA was controlled by spectrophotometry. The ratio of the optical density of 260 nm and 280 nm was not less than 2.

### Protocols of purification of the tight DNA-protein complexes

Two alternative protocols were used as internal controls. These are based on different principles, and DNA fragments of different sizes are generated in these methods.

### Isolation of TBP complexes with DNA using fractionation on nitrocellulose

DNA was digested with Hind III and Pst 1 restrictases or alternatively with Alu I restrictase (1U/1 µg of DNA) at 37 °C overnight in the appropriate buffer. Completeness of DNA digestion was tested by gel electrophoresis. The disappearance of the high-molecular DNA fraction and formation of the smear were criteria for the completeness of digestion. The restrictase-digested DNA sample was diluted in 3 ml of filtration buffer to a final concentration of 25–70 µg/ml. The solution was pressed through a nitrocellulose filter pre-soaked in a filtration buffer (HAWP 025 00, HA 0.45 µ, 25 mm, Millipore) supported in Swinnex filter holder. To avoid cross-contamination of the fractions, the filter was washed up to five times with the same volume (3 ml) of filtration buffer. The filter-retained DNA fraction enriched in the tightly bound proteins was eluted with 3 ml of elution buffer (five washes) and 3 ml of 50 mM NaOH in sequence (Werner & Neuer-Nitsche, 1989). The DNA content of the filtered fraction (F), low-ionic strength eluted fraction (R1) and alkali-eluted fraction (R2) were measured spectrophotometrically on NanoPhotometer NP80 Touch (Implen, Munich, Germany). A schematic presentation of protocols was published previously (Sjakste et al., 2009). Cloning was performed with the leaf F fraction DNA (FF), coleoptile R1 and R2 fraction and DNA extracted from the coleoptiles, leaves and roots (CR1, CR2, LR1, LR2, RR1, RR2 fractions respectively).

### Isolation of TBP complexes with DNA using exhaustive Dnase I digestion

The DNA was digested with DNase I (Fermentas) (1U/100 µg, room temperature, overnight) in 10 mM Tris–HCl, pH 7.6; 5 mM MgCl_2_. Completeness of digestion was monitored by gel electrophoresis. A faint band of about 100 bp was sometimes visible. To obtain the residual DNA fragments, the digest was incubated with Proteinase K (0.5 mg/ml) and SDS (0.5%) overnight at 37 °C and deproteinized with chloroform. Remnants of DNA were precipitated with ethanol (Avramova, Georgiev & Tsanev, 1994; scheme in Sjakste et al., 2009). DNA fragments were obtained from coleoptiles, first leaves and roots, with fractions called Cd, FLd and Rd, respectively.

**Cloning and transformation** were performed using pMOSB*Blue* Blunt Ended Cloning Kit (Amersham Biosciences, Piscataway, USA), as described by the manufacturer. Positive white colonies were picked and transferred to 100 µl of sterile water. Cells were lysed by keeping the tube in boiling water for 5 min. 10 µl of lysate was used for amplification with T7 promoter primer and U-19mer primer. PCR was performed using the Dream Taq polymerase (Fermentas, Vilnius, Lithuania) with the following parameters: 92 °C for 3 min; then 40 cycles 92 °C for 1 min, annealing temperature (55 °C) for 1 min, 72 °C for 2 min and a final extension step at 72 °C for 10 min. The presence of insertion was tested in the 2% agarose gel. The PCR product was purified using MinElute 96 UF Plates (Qiagen, Leipzig, Germany) and the NucleoVac vacuum manifold (Macherey-Nagel, Düren, Germany), according to manufacturers’ protocols.

**The sequencing** reaction was performed with primer M13 21uni and 5 µl of the purified product. Sequencing was performed on the MegaBace 1000 Sequencing System (Amersham Biosciences, Piscataway, USA).

### Computational analysis

EditSeq and SeqManII modules of the Lasergene expert sequence analysis software were used for sequence editing and assemble/contig management correspondingly. Blast search was performed using sequence databases at NCBI, CR-EST (Crop EST database at IPK), TREP (the Triticeae Repeat Sequence Database) and EnsemblPlants (http://plants.ensembl.org/Hordeum_vulgare/) databases. Comparison to MAR/SAR sequences was performed using http://smartdb.bioinf.med.uni-goettingen.de/cgi-bin/SMARtDB/smar.cgi database. Prediction of G quadruplexes (GQ) was performed with R-studio library pqsfinder.

### Circular dichroism spectroscopy of G-quadruplex forming sequences

Oligonucleotides corresponding to probable GQ-forming sequences were designed and ordered in Metabion (Table 1). Oligonucleotides were diluted in Milli-Q water to obtain a 100 µM stock solution. The stock solutions were incubated at 4 °C to achieve complete solubilization. Then, samples were prepared by dilution in folding buffer to a final concentration of 5 µM and annealed in the presence of the 100 mM KCl by heating to 95 °C for 5 min in a heating block and slowly cooling down at room temperature. Spectra were detected immediately and after 3 h. Samples were stored at 4 °C. CD spectra were recorded on a Chirascan CS/3D spectrometer (Applied Photophysics, Surrey, UK), 10 mm path-length at room temperature. CD spectra of DNA were recorded in a quartz cell off 10 mm path-length in a range of 340–200 nm, scan rate 200 nm min^−1^, bandwidth 1 nm, numbers of scan 4 averaged, at room temperature.

**Table 1:** Sequences of the oligonucleotides used for the CD experiments.

**Name**	**Sequence**	**Source**
Ct-2	5′-GGGAGAGGGAGAGGGAGAGGG-3′	DNase digested coleoptile DNA
Ct-11-2	5′-GGGGGGAGGGAGGAGAGAGAGAGAGAGGGAGAGGAGAGAGGGG-3′	R1 fraction from leaves
Ct-R1-6-2	5′-GGGGAGGGAGATGGAGAGAGGGAGAGAGGGG-3′	Coleoptile R1 fraction
68-LR1	5′-GGGAGGAGGTGGGAAGGGAGGG-3′	R1 fraction from leaves
44-LR1	5′-GGTGGAATCGGGGCCTAGAGCTCGGAATCGAGGGGGAGAAGAGGGGG-3′	R1 fraction from leaves

## Results

### Cloning

The tight DNA-protein complexes were purified from the first leaves, coleoptiles, and roots of barley seedlings using chromatography on nitrocellulose and exhaustive digestion of DNA with DNase I. Deproteinized and additionally purified DNA fragments (R1-DNA, R2-DNA after fractionation on nitrocellulose and DNA protected by proteins against DNase1) were cloned in Eco RV site of pMOSB*Blue* Blunt Ended vector. The cloning procedure of all the DNA samples was efficient. The blue/white screening enabled the identification of a large number of positive colonies. Bacterial lysates were prepared from the maximal possible number of positive colonies. From 48 to 140 DNA samples of every specimen were taken for the PCR-based insert amplification. PCR products were identified on the 2% agarose gels. All successful PCR products without size selection were used in sequence analysis. The purified PCR products were sequenced; the capacity of the experiment is given in Table 2. Altogether, about 477 DNA fragments were sequenced to produce a representative set of sequence complexity, according to the barley tissue studied, as well as the method of TBP fractionation applied.

**Table 2:** Capacity of thecloning and sequencing results.

Protocol	DNA specimen		Number of positive colonies	Number of PCR products	Number of sequenced inserts	% of sequenced clones	Range of fragment length	Average length (bp)	GC content
DNase I digestion	Leaves		316	137	97	30%	9–433	50	53%
		Coleoptiles		118	83	63	53%	9–438	111	58%
	Roots		121	74	41	34%	10–265	70	70%
Fractionation on nitrocellulose	Leaves	FF	140	43	39	28%	37–202	116	43%
				R1	174	99	57	33%	16–447	90	57%
			R2	165	112	52	31%	13–401	75	59%
		Coleoptiles	R1	93	93	36	39%	16–294	50	69%
			R2	80	80	32	40%	16–701	79	63%
		Roots	R1	133	88	41	31%	12–306	45	70%
	R2		134	48	19	14%	15–369	133	50%
	Sum		1,474	857	477				

### Statistical analysis of the sequences

The average length of the “free” DNA fraction inserts was 116 bp; average GC content was equal to 43%, very close to the average *Hordeum vulgare* DNA GC content (44.15%) published in the NCBI Genome database. The average length of the coleoptile R1 (CR1) fraction sequences was 50 bp, but the GC content was higher than in the genome (69%). Similarly, CR2 sequences were 79 bp long on average and GC-rich (63%). Clones of the retained DNA obtained from the leaves had an average length of 90 bp in LR1 fraction and 75 bp in LR2 fraction. The GC content was also high, 57% and 59%, respectively. In the root R1 fraction, GC content was even higher (70%) with an average length of 45 bp. However, the RR2 fraction did not follow this trend: it contained several AT-rich sequences, and the average GC content was still higher than in genomes (50%, average length—133 bp). Some differences were observed for clones obtained by exhaustive DNase digestion. In coleoptile, the average length of DNA-derived clones was 111 bp with an average of 58% of GC pairs. Clones obtained from the first leaves were mostly shorter (50 bp on average). Many were AT-rich, although GC-rich clones were also identified; the average GC content was 53%. The root clones were mostly GC-rich (70% on average), and the average length was 70 bp.

### The similarity of the sequences

BLAST alignment revealed homologous sequences in the barley genome for a majority of clones. However, no similarities were found for 7 clones derived from the “free DNA” (FF), 5 CR1 fraction clones, 10 CR2 clones, 13 LR1 and 16 LR2 sequences and 26 RR1 and 11 RR2 sequences. Among the clones obtained by exhaustive DNase digestion, 29 coleoptile-derived, 26 first-leaf derived and 12 root-derived clones did not align with the published barley sequences. Indeed, this is not surprising as the barley genome has not been completely sequenced. Initially, the sequences revealed by The International Barley Genome Sequencing Consortium (2012) represented about 80% of the whole genome. The following almost complete sequence did not represent 100% (Mascher et al., 2017). Our data give insight into sites difficult to sequence. Sequences without similarity in the databases (barley genome at NCBI and Ensembl) exceeding 30 bp in length were submitted to the NCBI database Sequence Read Archive (SRA). BioSample numbers are given in Table 3. In cases where we could identify our sequences from R1, R2 and DNase-resistant fractions in the well-characterized barley genome fragments, they were mostly localized in the repeats (Caspar, retrotransposons BARE 2-3; BARE_N15C-1, RLG_BAGY1_Y14573-1, Ashbury-2, gypsy, Sherlock, Athila Sabine, Wham-M19C-1; Wham_T14A-1; WhamD18-1-SoloLTR, Sabrina T8A1, LINE Morpheus, Sukkula_N15C-1, transposons CACTA and Balduin). That is not surprising, as 84% of the barley genome is formed by the repeats (International Barley Genome Sequencing Consortium, 2012). However, some clones aligned with both nuclear and chloroplast structural genes—several LR1 clones aligned with chloroplast psbA and psbE genes. Other clones aligned with ribosomal proteins L22 and S19, intron 1 of receptor like-kinase MybS3 gene, intron 2 VRN-H1 and some unidentified genes. Thus, the tightly bound proteins can anchor both DNA repeats and structural genes.

**Table 3:** Accession numbers ofthe non-aligned sequences in the NCBI database sequence read archive (SRA).

**BioSample**	**Type of the clones**	**Number of sequences**
SAMN13140880	R1 fraction of coleoptiles	15
SAMN13140881	FF fraction	9
SAMN13140882	LR1 fraction	11
SAMN13140883	LR2 fraction	13
SAMN13140884	RR1 fraction	24
SAMN13140885	RR2 fraction	12
SAMN13140886	DNA from the coleoptiles digested with DNaseI (Cd)	21
SAMN13140887	DNA from the first leaves digested with DNaseI (FLd)	16
SAMN13140888	DNA from the roots digested with DNaseI (Rd)	12

### Peculiarities of DNA sequences

The analysis of sequences forming complexes with tightly bound proteins revealed two specific motifs characteristic of this DNA fraction (Supplemental Information 1). These were CC(TCTCCC)n repeat, the so-called CT-motif found in18.9% of all inserts, and GCTCGTCCGGCTCGGAGGCTGACGATGGGCCCCGCCTGTCGGTCTCTCG sequence, the so-called GC-motif found in 6.9% of all inserts. These sequences were not found in any of the clones derived from protein-free DNA (F fraction). Sequence CC(TCTCCC)_2_TC was found in about 90% of all sequences containing the CT-motif. Other versions of the motif are presented in Supplemental Information 1. Interestingly, the CT motif contained AGAGG/TCTCC repeats. These 5-mer repeats were previously described as sequences characteristic of chicken DNA associated with tightly-bound proteins (Werner & Neuer-Nitsche, 1989). Blast search with CT-motifs produced more than 200 high-scoring results with barley ESTs Database. Blast against TREP (GrainGenes) revealed 25 blast hits including - retransposons LTR (athila, gypsy, copia, and others) and transposons (CACTA, mutator). Similar sequences were abundant in rice, tomato and *Arabidopsis* genomes. TCTCCC motif also gave multiple alignments with human and mouse genome sequences. GC motif gave 6 alignments with the barley EST database. It seems that a full 49-bp motif is composed of parts of different mobile elements including retransposons (gypsy, copia) and transposons (CACTA). Both components of the motif aligned with 9 sites of rice genome and some sites of *Caenorhabditis elegans, Mezorhizobum loti, Cryptococcus neoformis* and rat genomes. CT-motif is more frequently found in clones derived from leaf and coleoptile DNA compared to root DNA. GC-motif was mostly found in clones derived from the root R-DNA. Interestingly, parts of the CT motif of 7 to 8 nucleotides (TCTCCCT, CTCCCTC, CCTCTCCC ) aligned with several GQ-forming sequences from rice, Arabidopsis and Glycinia (Takahashi et al., 2012).

### Similarity to the MAR/SAR sequences

The relationship between TBP-bound DNA and nuclear matrix attachment sites (MARs) has been constantly discussed (Sjakste et al., 2012). To test similarities between our sequences and MARs, we have compared them to sequences deposited in the MAR/SAR database. Surprisingly, there were no alignments with the nitrocellulose-retained DNA fractions (CR1, CR2, LR1, LR2, RR1, RR2). However, some similarities were found in 5 of the 39 clones derived from free DNA. In TBP-bound fractions obtained by exhaustive nuclease digestion, some alignments were revealed in 6 of the 100 clones of coleoptile DNA, 8 of 63 clones of the first leaf DNA and 4 of the 41 clones of the root DNA.

### Predicted guanine quadruplexes

Taking the GC content of the sequences into account, we became interested in the formation of quadruplexes in these sequences. The analysis produced by the pqsfinder program revealed numerous potential quadruplex-forming sites in the sequences. Amongst 39 free DNA clones, seven possible GQ were found (0.18 per clone). In the TBP-enriched leaf fractions, there were more GQ-forming sites compared to the free DNA: in LR1 and LR2 clones, there were 0.28 and 0.26 GQ per clone, respectively. However, clones obtained from coleoptile and roots did not follow this trend: in CR1, CR2, RR1 and RR2 clones 0.13, 0.16, 0.09 and 0.15 GQ per clone, respectively, were identified. TBP-DNA complexes obtained by the exhaustive DNAse I digestion GQ sites were more frequent. In 75 sequences of Cd fraction (coleoptile DNA exhaustively digested with DNase I), 46 potential quadruplex-forming sites were revealed (0.61 per clone). In 44 clones obtained similarly from roots, there were 24 probable GQs (0.54 per clone) and 29 GQs in 101 sequences from the leaves (0.27 per clone). In 43 selected clones containing the CT-repeat only, 10 did not contain GQs. In some clones, there were 2 and even 3 sites. Thus, GQ frequency turned out to be 1 GQ per clone. Among identified sequences, possible GQs were found both in transposable elements, Sherlock, BARE 1 LTR-1, LTR-retrotransposon Ashbury-2, and some structural genes, Chloroplast psbA gene, intron 1 MybS3 gene, intron 2 “VRN-H1”.

### Determination of guanine quadruplexes

**Figure 1:**
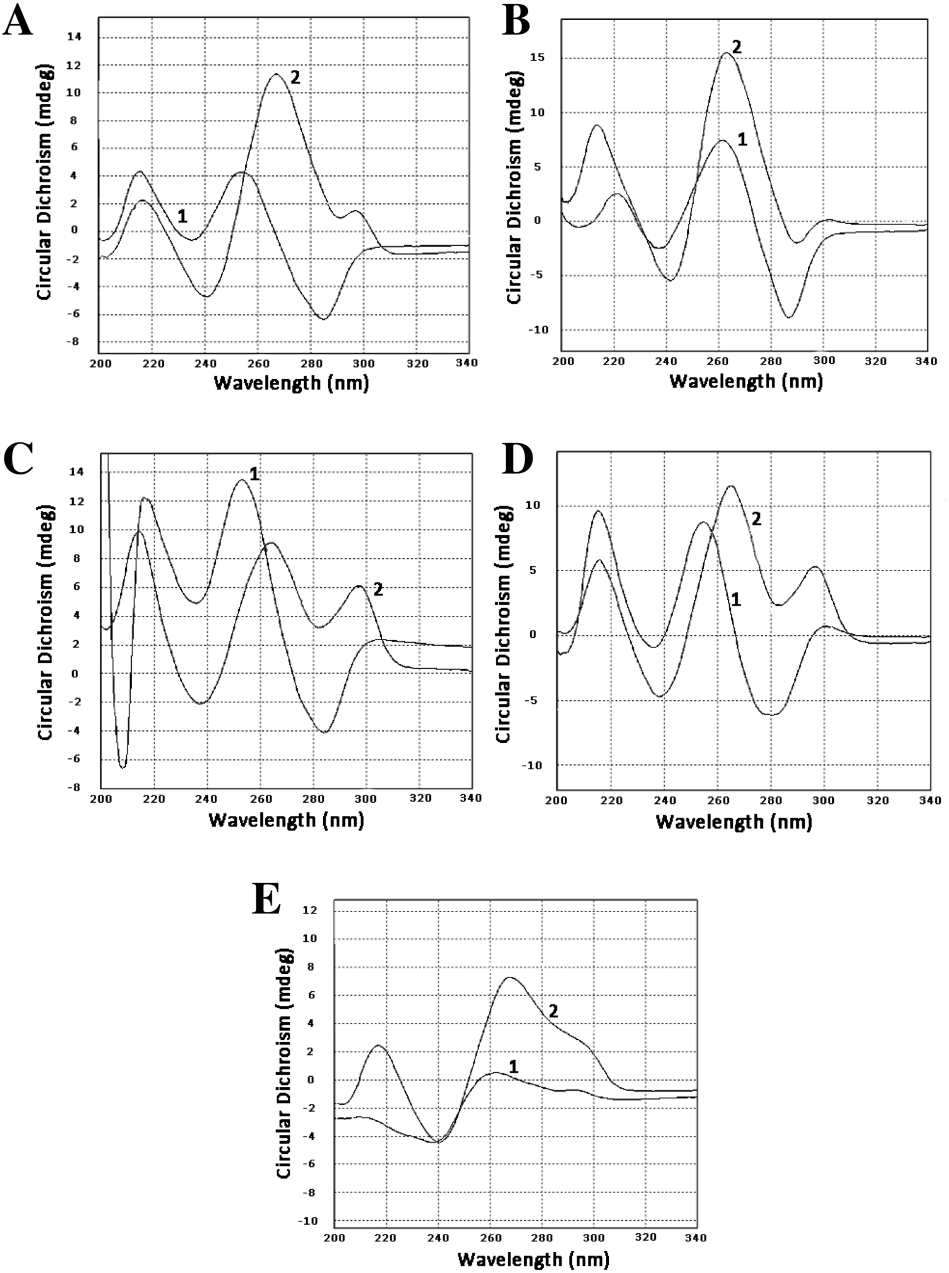
Circular dichroism spectra. (A) Oligonucleotide Ct-R1-6-2, 1—control, 2–3 h in 100 mM KCl. (B) Oligonucleotide Ct-11-2-F, 1—control, 2–3 h in 100 mM KCl. (C) Oligonucleotide Ct-2-F, 1—control, 2–3 h in 100 mM KCl. (D) Oligonucleotide 68-LR1, 1—control, 2–3 h in 100 mM KCl. (E) Oligonucleotide 44-LR1, 1—control, 2–3 h in 100 mM KCl. Raw CD spectra are given in the Supplemental Material.

To confirm that our sequences can form guanine quadruplexes, a set of oligonucleotides containing sites of possible GQs were designed and ordered. Three of them represented the minus strand of the CT-repeat. Two were derived from sequences of two clones of LR1 fraction and contained GC-rich motifs different from the CT motif. Circular dichroism spectroscopy revealed profound changes in spectra when oligonucleotides were incubated in 100 mM KCl. There was either an increase of positive band in the area of 260 nm (oligonucleotides Ct-R1-6-2, Ct-11-2-F, 44-LR1, Figs. 1A, 1B and 1E) or the formation of a positive band at 290 nm (oligonucleotides Ct-2-F and 68 LR1, Figs. 1C and 1D). In the former case, changes are typical for parallel G-quadruplexes, and in the latter, 3+1 structures (Lexa et al., 2014).

## Discussion

In the present work, we characterize the DNA part of tight DNA-protein complexes in barley seedlings. It should be mentioned that DNA was purified following a rather stringent procedure. Exogenous enzymes were not used to prevent the formation of artefactual DNA-protein complexes. Final DNA preparations were of high purity (A260/A280 = 2). Concerning the DNaseI protocol for purification of the TBP, the DNaseI resistance of the residual fraction is sooner due to the presence of the TBP. This was not due to the enrichment of the DNaseI resistant sequences, as we could not identify the most resistant DNA sites, the pure GC repeats (Lazarovici et al., 2013), in our clones. Moreover, this fraction is particularly protein-rich. The protein composition of these complexes have been characterized (Bielskiene et al., 2012), and the proteins turned out to be very diverse. However, several proteins involved in chromatin rearrangement and regulation processes, including transcription factors, serpins, protein phosphatases and protein kinases, RNA helicases, and DNA topoisomerase II, were identified. The DNA component turned out to be rich in GC and GQ-forming sequences, which were predicted bioinformatically. For some of them, the ability to form GQs was proven experimentally. We believe that the obtained spectra characterize mostly intramolecular GQs, parallel or of the 3+1 type, as the chosen concentration of oligonucleotides and time of incubation with KCl were not sufficient for the formation of intermolecular GQs (Zhou et al., 2014). A common CT-motif shared by numerous sequences involved in the tight complexes and capable of forming GQs was also revealed. The above findings could explain the presence of numerous transcription factors in the protein part of tight complexes, as in plant genomes. GQs are formed in the vicinity of the transcription start (Takahashi et al., 2012), in cereals like maize (Andorf et al., 2014) and rice (Wang et al., 2015a). GQs are found in 5′-UTR and at the 5′-end of the first intron in many genes, the transcription factor binding sites. In the LTR, retrotransposons GQs are also involved in transcriptional regulation (Lexa et al., 2014). GQ-binding proteins are poorly characterized in plants. A detailed study of these proteins is considered an important task (Griffin & Bass, 2018; Yadav et al., 2017). Plant nuclear protein extracts manifest a high affinity to oligonucleotides-forming GQ (Garg, Aggarwal & Thakkar, 2016). However, the ZmNDPK1 nucleoside diphosphate kinase 1 appears to be the only revealed GQ-binder in maize. The ability to bind these motifs can be predicted for RecQ family helicases (Griffin & Bass, 2018; Yadav et al., 2017). Interestingly, both kinases and helicases were identified among barley TBPs (Bielskiene et al., 2012). The formation of tight complexes by GQs in the regulatory regions of genes can contribute to the maintenance of the 3D network which is believed to be the form of genome folding (Razin & Vassetzky, 2017; Razin & Gavrilov, 2018). GQs could also determine the high affinity of DNA isolated from complexes with TBPs to proteins, as complexes can be reconstituted *in vitro* (Bielskiene et al., 2012). Recently, it was shown that the GQ position in genome co-localizes with TAD borders. Moreover, GQ overlaps with CCCTC binding protein sites (Hou et al., 2019). CCCTC protein binding and cohesion are considered to be main proteins stabilizing TADs in animals (Luzhin et al., 2019). Plants do not possess CCCTC binding protein (Wang et al., 2015b); chromatin packaging also differs from the animal model (Dong et al., 2017). However, the CCCTC sites can also be occupied by other proteins (Kaaij et al., 2019). The revealed CT-motif resembles the CCCTC binding factor site. Perhaps, some of the TBPs could also stabilize TAD borders. Of course, in our study, we could not prove the association between individual TBPs and GQs. This should be done in a special study, taking into account the great diversity of TBPs in barley (Bielskiene et al., 2012), a difficult task indeed.

We have also found out that DNA sequences of the tight DNA-protein complexes either do not contain MAR/SAR sequences or contain a minimal number of MAR/SAR sequences. Artefactual or not these AT-rich sequences are believed to anchor DNA to the nuclear matrix, forming a kind of tight DNA-protein complexes. The question of whether the deproteinization-resistant proteins were components of the nuclear matrix was intensively discussed (see Sjakste et al., 2010; Sjakste et al., 2012 for review). The present results indicate drastic differences between the DNA components of the nuclear matrix and TBP complexes. The former are AT-rich, and the latter GC rich.

Our results support the idea of the functional significance of tight DNA-protein complexes. Further studies could elucidate the role of individual proteins of the fraction.

## Conclusion

As initially assumed, DNA fragments are specific. These are GC rich sequences with multiple possible sites for G-quadruplex formation. Similarity to MAR/SAR sequences is absent or negligible. The frequent CC(TCTCCC)_2_TC motif can form G-quadruplexes *in vitro*. It is assumed that GQ sequences can trap proteins and stabilize the structure of the topologically-associated domains.

##  Supplemental Information

10.7717/peerj.8569/supp-1 Supplemental Information 1 Sequences characterized by the presence of the TCTCCC motif and their abundance infractions analysed Consensus motif TCTCCC is underlined, CCCTCT is marked in bold, variable 5′- and 3′-ends of the sequences are italicized.

10.7717/peerj.8569/supp-2 Supplemental Information 2 Raw CD spectra

## Additional Information and Declarations

### DNA Deposition

The following information was supplied regarding the deposition of DNA sequences: The sequences are available at NCBI Sequence Read Archive (SRA): SAMN13140880, SAMN13140881, SAMN13140882, SAMN13140883, SAMN13140884, SAMN13140885, SAMN13140886, SAMN13140887, and SAMN13140888.

### Data Availability

The following information was supplied regarding data availability: Raw CD spectra is available as a Supplemental File.
