# Supplementary material for: Tight DNA-protein complexes isolated from barley seedlings are rich in potential guanine quadruplex sequences
Source: PeerJ. 2020 Feb 18;8:e8569. doi: 10.7717/peerj.8569 (PMC7034378; doi:10.7717/peerj.8569)
Supplement: Supplemental Information 1 — Consensus motif TCTCCC is underlined, CCCTCT is marked in bold, variable 5′- and 3′-ends of the sequences are italicized. [file peerj-08-8569-s001.docx]

| Details on DNA- TBP complexes fractionation | |  |  |  |  |
| --- | --- | --- | --- | --- | --- |
|  |  | DNase I | Nitrocellulose fractionation | | |
| Sample | | Pooled data | First leaf | Coleoptile | Root |
| Number of clones sequenced | | 201 | 148 | 68 | 60 |
| Clone  name | Sequence of the fragment |  |  |  |  |
| CT-1 | *C/G*CC(TCTCCC)_2_T*C/CC* | 2 | 70 | 44 | 1 |
| CT-2 | CCC(TCTCCC)_3_TCC*T* | 2 |  |  |  |
| CT-3 | CCC(TCTCCC)_2_(TC)_4_CC | 1 |  |  |  |
| CT-4 | CCC(TCTCCC)_2_CCT | 1 |  |  |  |
| CT-5 | *CCT*CTC(TCTCCC)_2_*T* | 2 |  |  |  |
| CT-6 | CTCA(TCTCCC)_5_T**CCCTCT**CCTTCCCTGCCC | 1 |  |  |  |
| CT-7 | CTCCC(TCTCCC)_5_TCTCC | 1 |  |  |  |
| CT-8 | CTCCC(TCTCCC)_2_TCTTCCCCTCCCT**CCCTCT**ACC | 1 |  |  |  |
| CT-9 | *TCTCC*TCTCCC*TCTC(T)CT* | 3 | 6 |  | 1 |
| CT-10 | T**CCCTCT**CTCTCCCT | 1 |  |  |  |
| CT-11 | TCTCCCTCCCCTCTCTCCCTCTCTCCTCTCCCTC**CCCTCT**CTCCTCTCCCTCTCTCTCTCTCTCCTCCCTCCC**CCCTCT**CTCAATCTCCCTCT |  | 1 |  |  |
| CT-12 | CCTCTACTCTCCCTCCC(TCTCCC)_2_TCCCTCTCC | 1 |  |  |  |
| CT-13 | CCCTCCCCC(TCTCCC)_2_TCTCTCCCTCTC | 1 |  |  |  |
| CT-14 | CCTCTCCCTCTCTCCCTCTCTCC | 1 |  |  |  |
| CT-15 | ATATCCC**CCCTCT**CTCTCTCCCT | 1 |  |  |  |
| CT-16 | CCACTCCTCTCCCGCG | 1 |  |  |  |
| CT-17 | CCTCTCCCCTCTCCT |  |  |  |  |
| CT-18 | CCCCCTCTCCCTCTCTCCCCC | 1 |  |  |  |
| CT-19 | CCCTCTCCCCTCCCTCC**CCCTCT**CTCTCTCCCTATCTCTCTCTCTTTGCCTGTCTCTCTCC**CCCTCT**CTCTCTCT**CCCTCT**CTCTCTCCCTCTCCTCT |  | 1 |  |  |
| CT-20 | CTCTCTCCGCTCTCCTTCTCACTCTCCCTCTCTCCCT**CCCTCT**CTCCCCCTTCTCTCTCCCTC |  | 1 |  |  |
| CT-21 | CCGATTCTCCCTCGCCGCCGTCGCCTCCCGGAGCCGCAGACCGC | 1 |  |  |  |
| CT-22 | CTTCTTCCTCCGGCCGCCGGACCTCTCCCGTCGATTCGTCGGCCCCAGCCTTCCCCGAGCCGTCTGAGCTCTTCTCTGCACTCC | 1 |  |  |  |
| CT-23 | CTGCTTGGGTGTGGGAAATCGGTGGGTTTGCATATGAAATCATATGCAAACCTCCCGTTTCTCCCGTAACCCTTGCTTTTCCCAAACGTTGGCTCGGATGTCCCGTCGTTCTCCTGTCCCGTGTACGACTCATGCCAAATTCTGATCCGTCGGTCGAACGGCTGTTCGGGTTGCGAGAAAAGTAC | 1 |  |  |  |
| CT-24 | ATCCCTCTCTCTCTCCCCCTCTCCCTCCCTCCCTCTCTCTCCCTCTCT | 1 |  |  |  |
| CT-25 | CCTCTCCCTCTCCGTCTCCTCTCCCCTCTCTCTCTCGCG |  | 1 |  |  |
| CT-26 | CCTCTCCTCTCCCTCCCCTATAGTGAGTCTATTAAT |  | 1 |  |  |
| CT-27 | CCTCTCCCTCCTCCTCCTTCTTCTTCCTATTCTTCTTCTTCTTCTTCTTCTTC | 1 |  |  |  |
| CT-28 | CTTCTCCCATTGCAAGAATTATAGATC | 1 |  |  |  |
| CT-29 | ACAAACATGTGTGAAGATTATGAAAAGTTTCATGAAATTTCTCCCTTCAAAGTGAAGGCTAACCCAAGCTCCCAGGAGAGCGATGAAGATT | 1 |  |  |  |
| CT-30 | GCCTTCTCCCCAAGCACAGTCCGCTGGGTTCCTACTCCTCGATCCAGGCGGGATCGAGGGAGCGCGAGCCGCGGGTTCC |  | 1 |  |  |
| CT-31 | CTACTAGTCATATGGATCCCTCCCCCTCCCCCTCCCTCTCCCCCTCTCTT | 1 |  |  |  |
| CT-32 | CCTCTCCTCTCCT |  | 1 |  |  |
| CT-33 | CTTCCTCCCT**CCCTCT**CC | 1 | 1 |  |  |
| CT-34 | T**CCCTCT**CCT | 1 |  |  | 1 |
| CT-35 | CATCATCTCCTCCCT |  |  |  | 1 |
| CT-36 | TCCTCCTCCTCCTCCC | 1 |  |  |  |
| CT-37 | TCCCCCTCCCTCCACCTCCTCCTCTTCTTCCTCTTC | 1 |  |  |  |
| CT-38 | CTTTACTTTATTTTGAATCTTTTTTTTCTTCTTAATCTTTCTATTCAGAATTCAGTTAACGACGAGATTTAGTATCCTTTCTTGCATTTTCATAACTCGTAAAATGCCGAGTAGGCACGAATTCTCCCAATTTGCGACCTACCATAGGATTTGTTATGTAAATAGGTATATGTTCCTTTCCATTATGAATCGCAATTGTATGGCCAACCATTGTGGGTAGAATGCTAGATGCCCGGGACCACGTTACTATTGTTTCTTTCTCCTCCTTCATATTGACC |  | 1 |  |  |
| CT-39 | GTAATAAAAAGCCTTATAGTAAAGAATATGCCAGGACCTGATGATGGCTTCAATGCTGAATTCTAACGAACAGTGTACCATCTGAACCATTTCTTAAGTGTATAGTTCAGTGGTATNAAATATATTCATAATGACATGCAACCATCACCACCATCCATCTCTACAACTCTGTACCTAGCACACAATCACTCCCCATTCTCCATTCCCCCGACCCCAGCCCTTGGTAGCCTTCATTCTACTTTGTCTCTATGATTTTGACTCATTCTACCTCATAAAAGTGGAACCACGGAATATTTGCCTTTTTGTGACTGGCTTATTTCACTCAGTGTAATGTCCTCCAGGTTCATCCATGTTGTAGCATGTGTCAGAATCTTCTTTCTTTTTTTTGAGATGGAGTCTTGCTCTATCTCCCAG |  | 1 |  |  |
| CT-40 | TCCTTCTCCTCCTCCTCCTCCTCCTCCTCCTCCTCCTCCTTCTTCTTCTTCTTCTTCTTCTTCTTCTTCTTCTTCTT |  |  |  |  |
| CT-41 | CGTGAGCTCAGGGTGCTTTTCC**CCCTCT**TCTCCCCCTCGATTCCGAGCTCTAGGCCCCGATTCCACCGGAGCCCGACGAGATCCGCCGCGGGAGCTCATCGTCGGTATGCTCCGATGATCGGTTGGTCGTTCTGAGGTCACCGTCGGTCCAGCTCGTCGCGTAG |  | 1 |  |  |
| CT-42 | TGCATATGTGCCCTACGTGTTGGACAAGTTTTACTGGTCCTAGTGGCCCTGTGGACATGAGTGGCCATGGTGACCGCGCTGGCAACAGGTAGTAGCGGTGCTCTCCACCCTTTCTTCGTCCTATAGCTTGGGTCCCTCAACGGCCGCGTGTCCCAGCGCGCCGGGGCACCTCCCTTGACCAGTCCAACCCATCCCGACGCCTAGAAACAGCCAAATATCCCGTGGCACGCGTTCTATCGCCGTGCACCGAAGCTCCAACAGCTCTCGAGCTTTCTCTTCCTCTCCCCCTCTGCTTCCTCTTCCTCTTG | 1 |  |  |  |
| CT-43 | CCTCCTCGTGTGATAGGGATGAGGGGCACGGCGGCGGCAGTGGTGCCCCTGGCAGGTCAGGATCCCCAGTGGTCAGGTCGGAGTACGATTCCCCGGGTTCTGACTGACCATCGGCCCTCCCACCGAGTCCCGCTCACGCCTCTCCCCCGCCCGACGGGGGTATCTTTCGGAAAGTCGTGCCCGCGGTTATGTGGTAGACTTGGAAACTTTTTTTAACATACGGTTCTAGATAACTTGAAGTACACTTAATTAAAGACTTGCCAACTGAGTGCGTAACCGTGATTGTCTCTTTCG | 1 |  |  |  |
