# Supplementary figures and images for: Tight DNA-protein complexes isolated from barley seedlings are rich in potential guanine quadruplex sequences
Source: PeerJ. 2020 Feb 18;8:e8569. doi: 10.7717/peerj.8569 (PMC7034378; doi:10.7717/peerj.8569)

## Slide 1
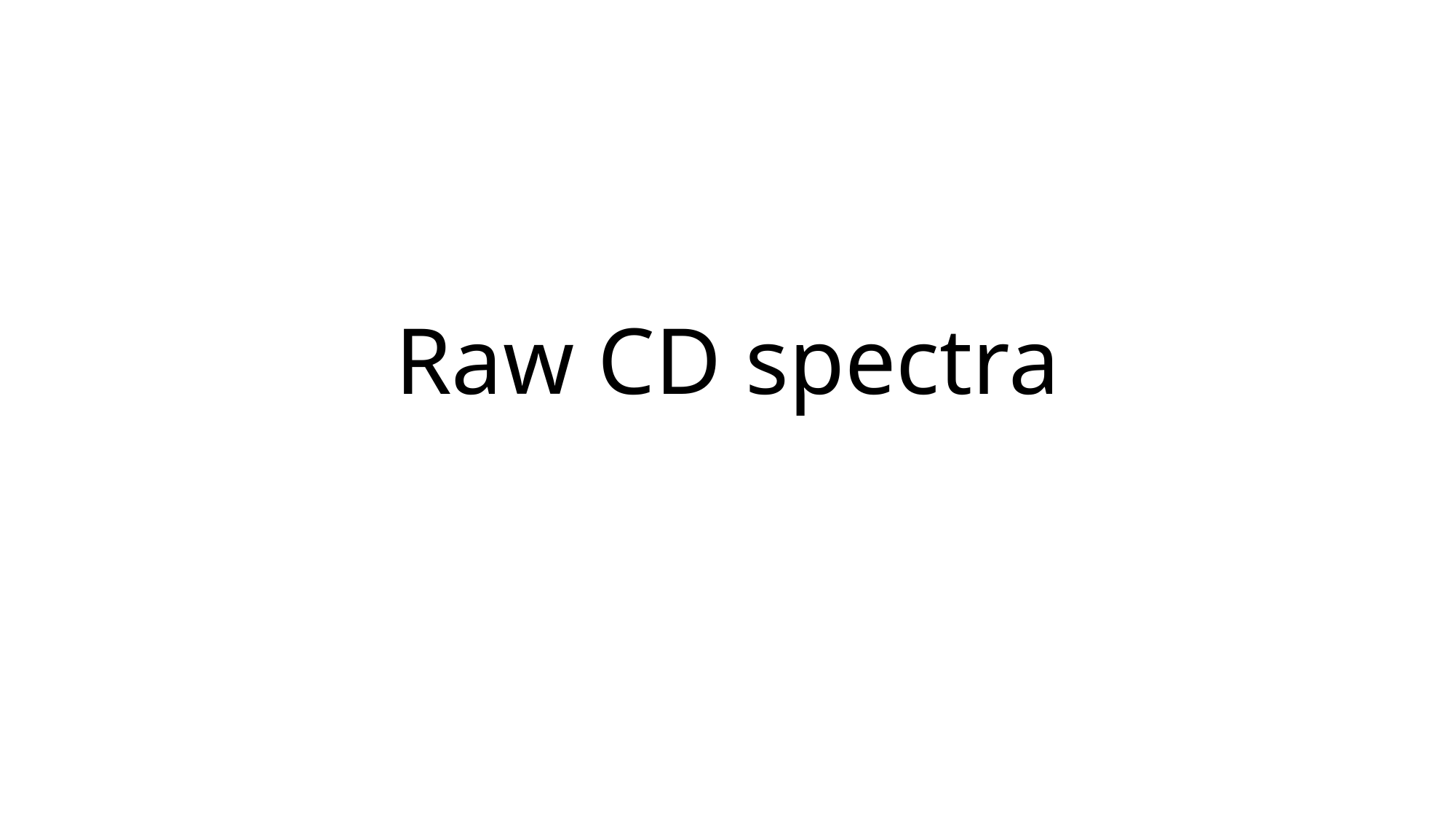

# Raw CD spectra

## Slide 2
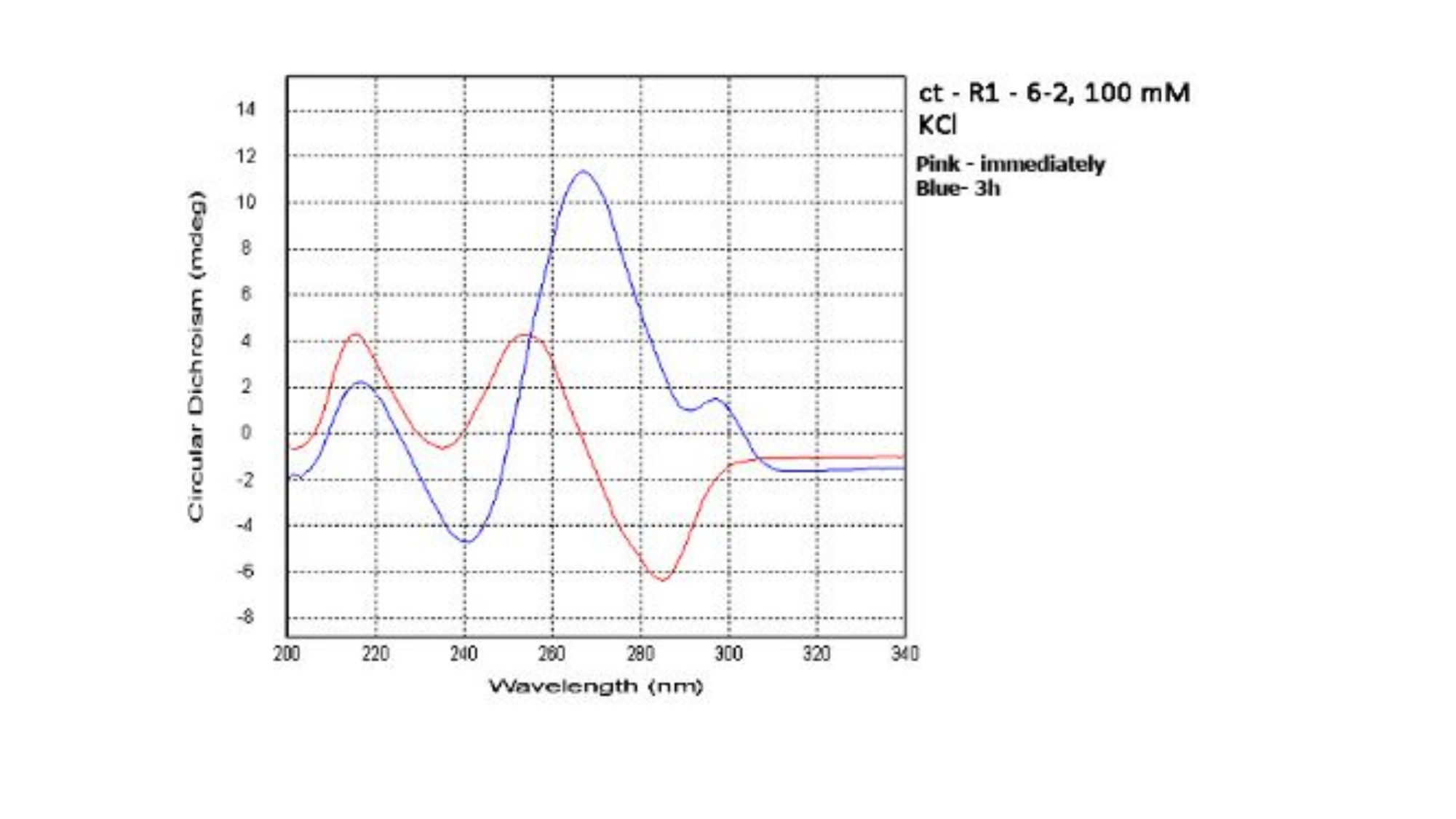

#

## Slide 3
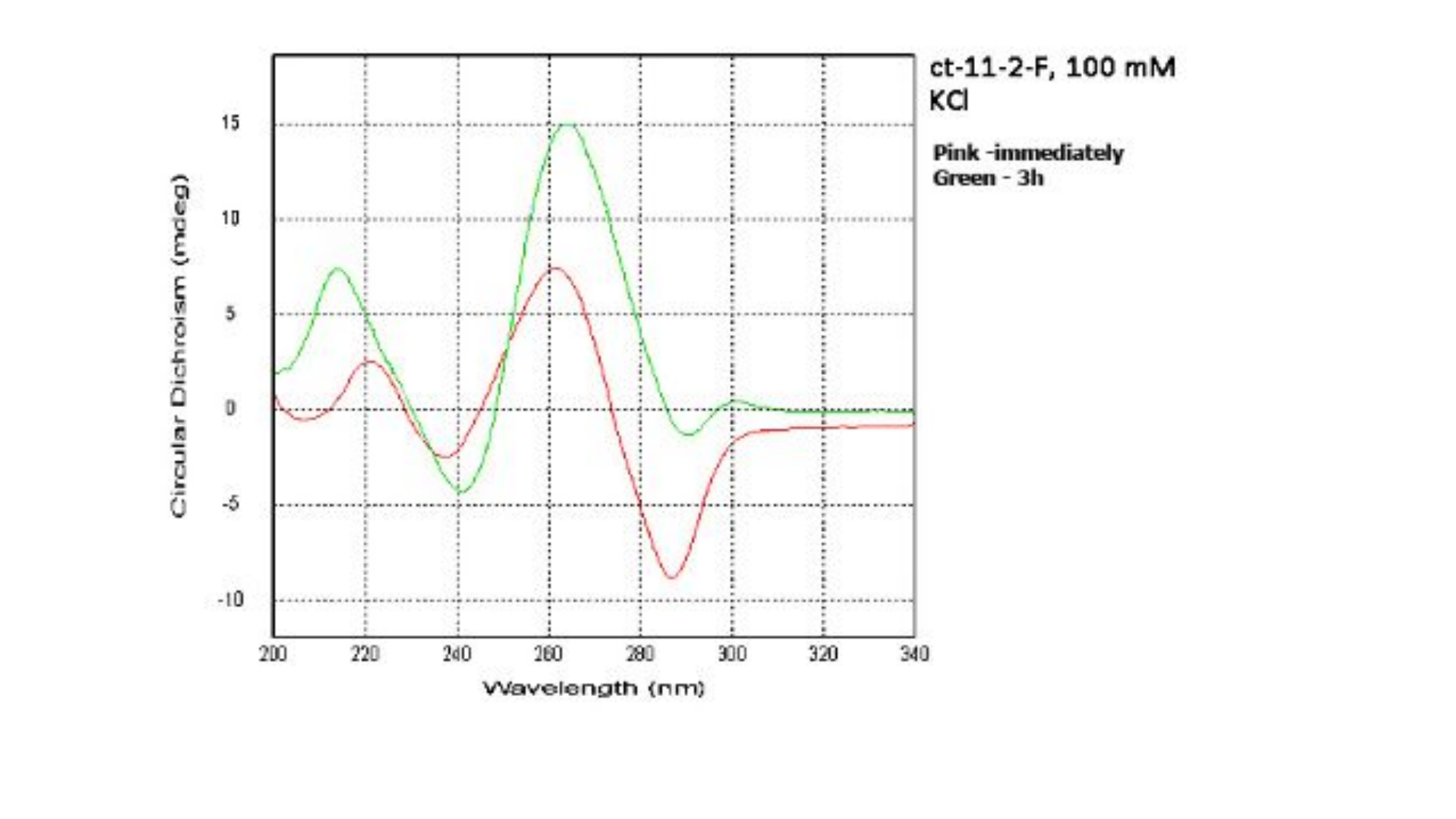

#

## Slide 4
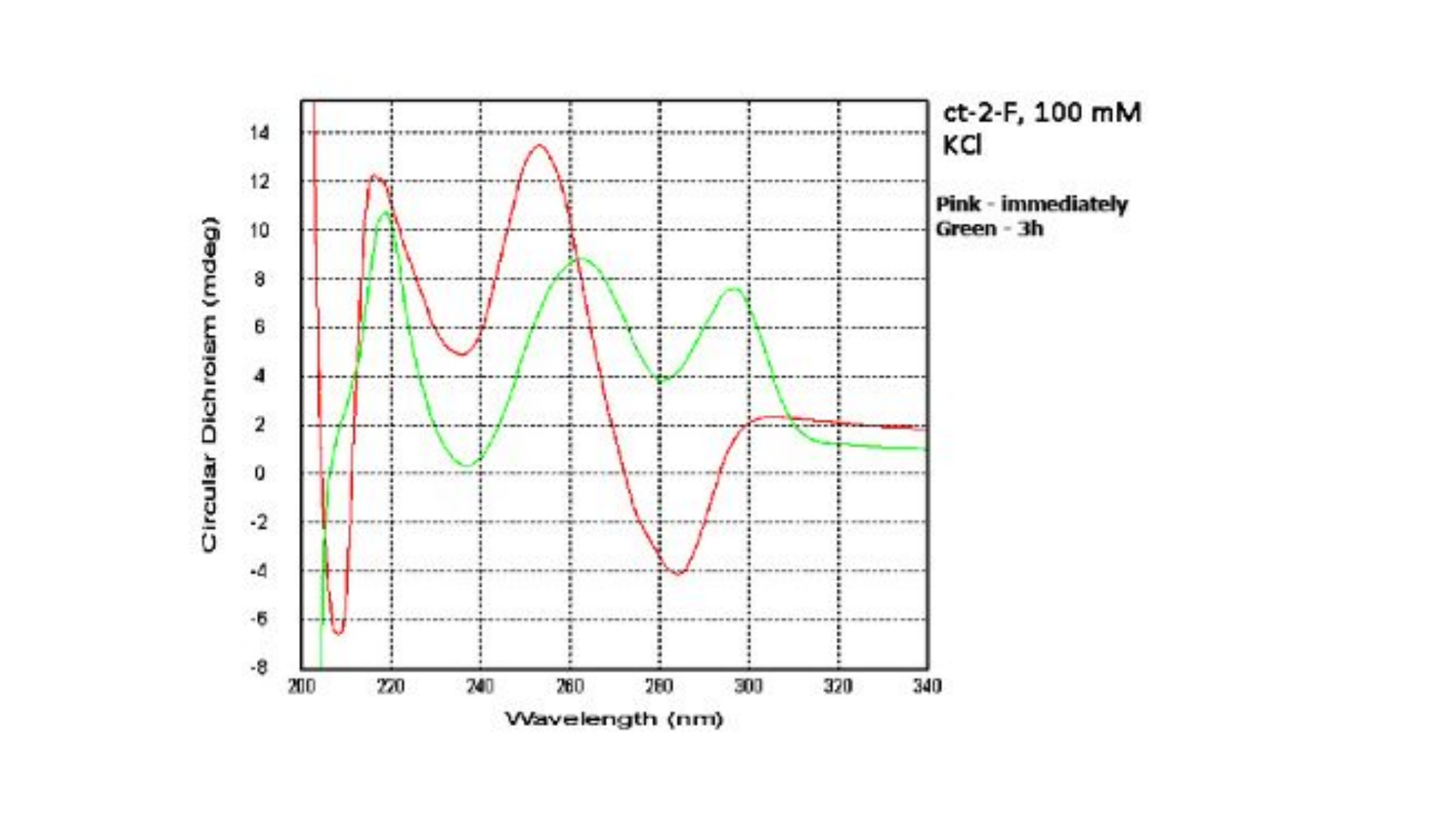

#

## Slide 5
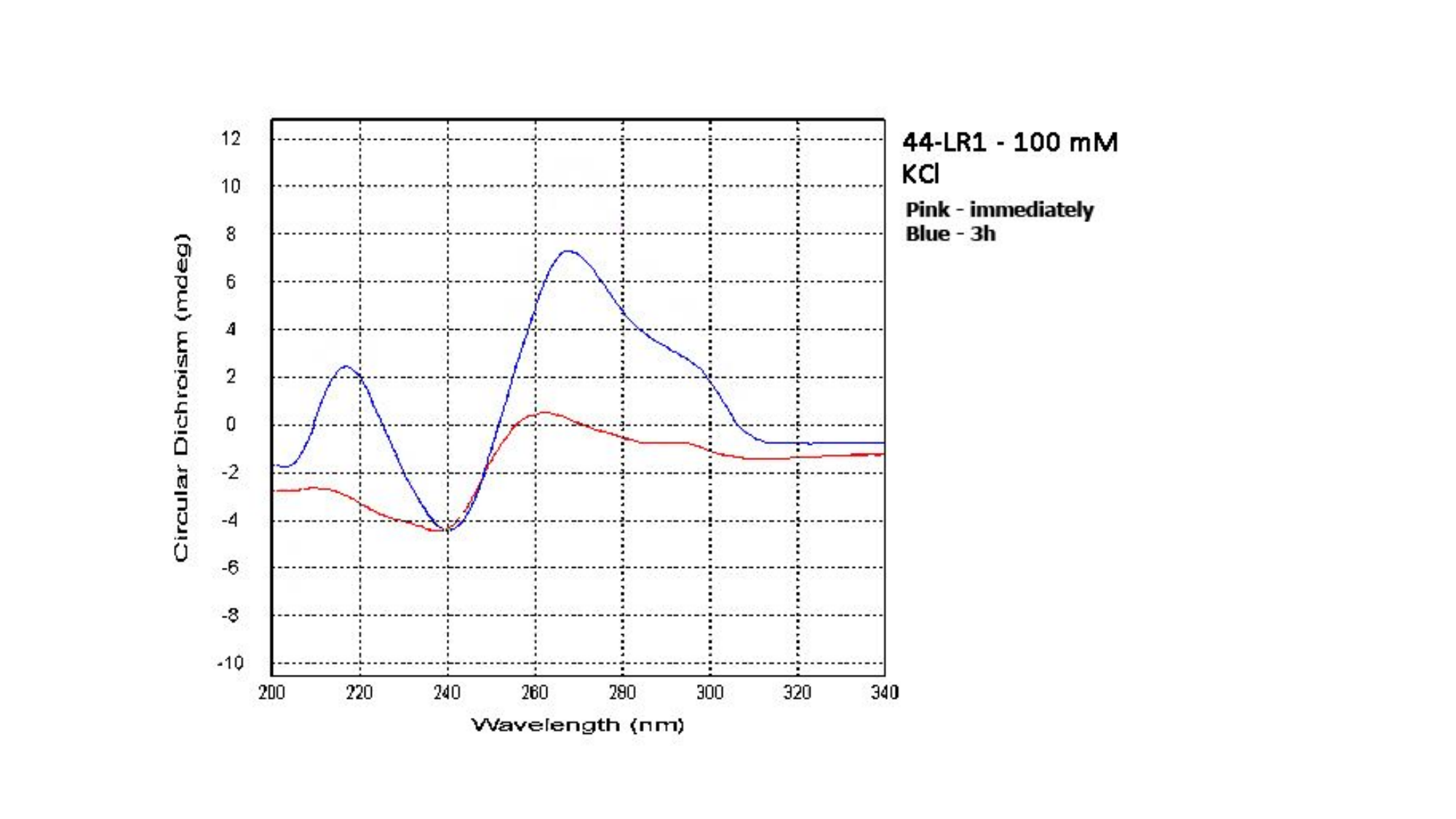

#

## Slide 6
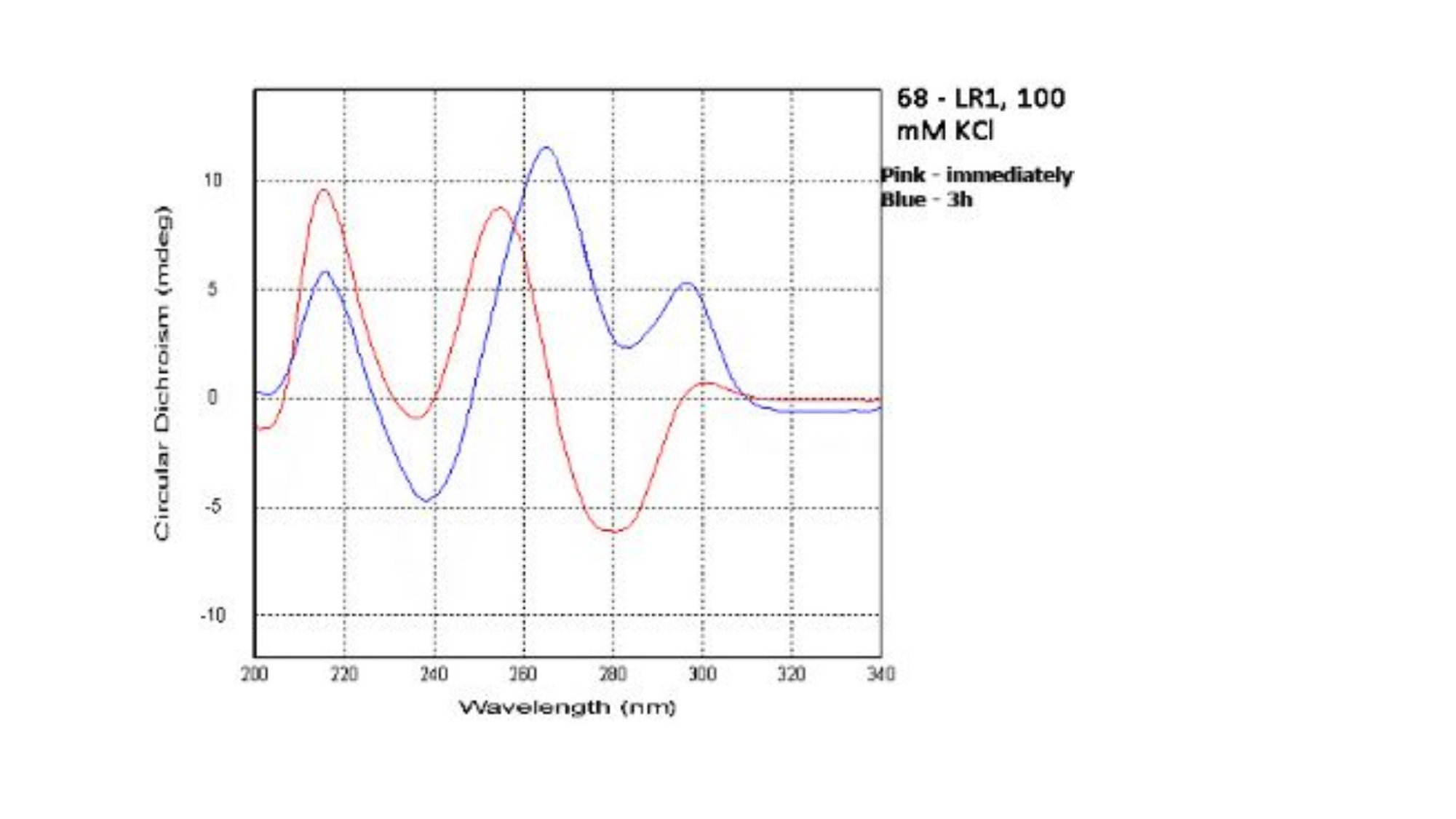

#

Supplement: Supplemental Information 2 [file peerj-08-8569-s002.pptx]
